# Puerperal Fevers

**Published:** 1890-02-22

**Authors:** 


### PUERPERAL FEVERS.

Mr. R. D. Pedley, dental surgeon to the Evelina Hospital, contributes a paper on a possible source of contagion of puerperal fever (*Lancet*). The author asks is it possible for a medical practitioner, or a nurse, whose mouth is in an unhealthy condition to be the means of developing puerperal fever, or any of the local manifestations of septic poisoning, such as pelvic cellulitis or pelvic peritonitis? Chronic alveolar abscesses from diseased teeth are common, and the discharge is unpleasant to both taste and smell. It is not unreasonable to suppose a nurse may seek to relieve pain of an aching stump by pressure of the fingers, and thus convey septic matter to a patient. The author cites Dr. Playfair, who mentions the case of a medical man suffering from chronic ozæna who had to relinquish practice on account of the numerous deaths from puerperal fever among his patients. He also refers to a recently-reported inquest, where an uncertified midwife attended a case of puerperal fever, having been previously treated herself for a piece of dead bone in her mouth. In May and September, 1886, two cases came under Mr. Pedley's notice where a nurse and a medical man, suffering from alveolar abscess had puerperal fever cases. If, however, puerperal fever is communicated in this manner it is more likely to be so through the nurse than through the medical man, as the former is brought more constantly in contact with the patient. Moreover, a medical man may be trusted to do his best to avoid injury by the use of antiseptic precautions. In conclusion, the author thinks that it is desirable midw ves and nurses should be subjected to medical inspection before attending obstetric cases. Mr. Pedley's observations demand attentive consideration, for cases are on record of patients who have died from pyœmia and septicœmia as the result of alveolar abscess.

